# Cancer pain awareness and communication practices among physicians in China: a nationwide mixed-methods study

**DOI:** 10.1038/s41598-026-43569-7

**Published:** 2026-03-12

**Authors:** Shasha Shen, Dahai Liu, Xiaotong Shen, Sisi He, Xiao Liu, Hui Xu, Kai Zhou, Yu Zhang, Xiaoxia Gou

**Affiliations:** 1 Department of Head and Neck Oncology, The Second Affiliated Hospital of Zunyi Medical University, Zunyi, Guizhou China; 2 Department of Radiation Oncology, The Second Affiliated Hospital of Zunyi Medical University, Zunyi, Guizhou China; 3 Department of Nuclear Medicine, The Second Affiliated Hospital of Zunyi Medical University, Zunyi, Guizhou China; 4 Department of Thoracic Oncology, The Second Affiliated Hospital of Zunyi Medical University, Zunyi, Guizhou China; 5 Department of Abdominal Oncology, The Second Affiliated Hospital of Zunyi Medical University, Zunyi, Guizhou China

**Keywords:** Cancer pain, Pain management, Physician knowledge, Assessment tools, Opioids, Multidisciplinary care, China, Mixed-methods study, Cancer, Health care, Medical research, Oncology

## Abstract

Effective cancer pain management remains a global challenge, often hindered by gaps in physician knowledge, attitudes, and institutional awareness. This study aimed to assess Chinese physicians’ understanding, perceptions, and practices related to cancer pain management through a national mixed-methods survey and interview study. A nationwide mixed-methods study was conducted between September 2019 and September 2024 to assess cancer pain management practices. The quantitative component involved a large-scale online survey distributed across 29 provinces to capture broad trends in physician knowledge and clinical behavior. To ensure a comprehensive evaluation, the study integrated a qualitative component through semi-structured interviews with frontline clinicians, aiming to gain deeper insights into unmet clinical needs and systemic barriers. A multi-step analytical approach was used to examine the relationship between physician demographics—such as education and specialized training—and their pain management awareness. Among 2,188 respondents, the majority were early-career physicians at tertiary hospitals. While a high percentage recognized the multi-dimensional nature of cancer pain, including sensory and social factors, practical assessment relied heavily on rapid subjective scales. Quantitative analysis demonstrated that higher educational levels and specific clinical exposure, such as oncology department rotations, were significant predictors of stronger pain management awareness. Conversely, a reliance on non-opioid medications was associated with lower awareness levels. Qualitative findings corroborated these trends, revealing a predominant reliance on pharmacological interventions and a critical need for structured multidisciplinary training. This study identifies potential knowledge gaps and variability in cancer pain management practices among physicians in China. The findings suggest that targeted training programs, interdisciplinary collaboration, and the standardization of assessment tools may be beneficial to improve clinical outcomes and patient quality of life. Furthermore, these results underscore the need to address resource disparities to promote more equitable pain care across different regions.

**Supplementary Information:**

The online version contains supplementary material available at 10.1038/s41598-026-43569-7.

## Introduction

Cancer pain is one of the most common and distressing symptoms experienced by patients with malignancies, significantly affecting their quality of life and functional status^1,2^. Despite advances in oncology, effective pain control remains a global challenge due to the complex and multidimensional nature of cancer pain, which involves sensory, emotional, cognitive, and social aspects^3,4^. Comprehensive assessment and multidisciplinary management are key to improving outcomes, yet gaps in clinical practice persist worldwide. Comprehensive assessment and multidisciplinary management are key to improving outcomes^5^. In this study, ‘Multidisciplinary Management’ is defined not merely as a theoretical concept, but as a framework for integrating pharmacological, psychological, and physical therapies. Our analysis treats it as both a perceptual attitude among clinicians and a measurable clinical deficiency, highlighting the gap between the high recognition of its importance and the current predominant reliance on single-modality pharmacological interventions^6–8^.

In China, uneven economic development and healthcare resource allocation have resulted in disparities in pain management across different regions^9,10^. Physicians’ characteristics such as education level, clinical experience, and specialty background influence their understanding and implementation of cancer pain management strategies^11^. However, limited data exist on the nationwide status of physician awareness and practice in this area.

Globally, numerous studies have highlighted barriers to optimal cancer pain control, including inadequate knowledge among healthcare providers, limited use of standardized assessment tools, and underutilization of non-pharmacological interventions^12,13^. International guidelines emphasize the importance of routine pain assessment using validated scales and individualized, multimodal analgesic regimens^14^. Nevertheless, real-world adherence to these recommendations varies.

Previous reports from China have shown predominant reliance on pharmacological treatment, mainly oral opioids, while non-pharmacological and psychological approaches are less frequently applied^15^.Addressing these gaps through targeted training and resource allocation is essential to standardize care and improve patient quality of life. In China, the management of cancer pain is deeply intertwined with cultural beliefs^16^. Many patients adhere to a ‘stoic’ philosophy, viewing pain endurance as a sign of character, which leads to significant underreporting^17^. Simultaneously, ‘opiophobia’—the irrational fear of addiction—remains a major barrier among both the public and healthcare providers, often resulting in the underutilization of standardized opioid titration even when clinically indicated. Addressing these cultural stigmas is essential for implementing effective pain management protocols^18,19^.

This study aims to provide a comprehensive evaluation of physicians’ knowledge, attitudes, and practices regarding cancer pain management across mainland China, and to explore factors associated with pain management awareness. The findings may guide future educational programs and policy initiatives to enhance cancer pain care nationally.

## Methods

### Study design

This study adopted a cross-sectional design to assess Chinese frontline clinicians’ knowledge and practices regarding cancer pain. The mixed-methods approach was chosen to integrate the statistical breadth of nationwide quantitative data with the contextual depth of qualitative clinical insights. This study employed an explanatory sequential mixed-methods design. Data triangulation was achieved by using qualitative interview findings to contextualize and explain the statistical patterns identified in the quantitative phase. Specifically, the qualitative data helped elucidate the systemic and cultural barriers that contribute to the gaps in physician awareness revealed by the survey. Physicians from multiple specialties—including oncology, internal medicine, surgery, and pain management—were recruited nationwide through convenience sampling between September 2019 and September 2024; this extended period was utilized to ensure a robust and representative sample across 29 provinces despite the logistical challenges of large-scale data collection.

### Quantitative survey

A structured, self-administered questionnaire consisting of 36 items was developed based on established clinical guidelines and existing literature to collect information on demographic characteristics, understanding of cancer pain, and clinical management practices. A pilot test was conducted with 30 participants to refine the language and ensure reliability before formal distribution. The survey was distributed via the online platform Sojump.

### Participants and data collection

To ensure the authenticity and professional status of the respondents, the survey was distributed via the professional platform Sojump, which required participants to specify their hospital affiliation and professional title. Only practicing physicians were eligible to submit the response. Furthermore, a pilot test was conducted with 30 clinicians selected through convenience sampling from the Second Affiliated Hospital of Zunyi Medical University. These participants were chosen based on their clinical experience in oncology and pain management to ensure the questionnaire’s face validity and linguistic clarity before national distribution.

### Qualitative interview

Semi-structured in-depth interviews were conducted with a purposive sample of 10 frontline clinicians. The sample size was determined by data saturation, where no significantly new themes emerged in the final interviews. Qualitative data were analyzed using thematic analysis to identify key perspectives on clinicians’ definitions of cancer pain, clinical decision-making, and training needs.

### Statistical analysis

All statistical analyses were conducted using IBM SPSS Statistics version 29.0 (IBM Corp., Armonk, NY, USA)11. Descriptive statistics, including frequencies, percentages, means, and standard deviations (SDs), were used to summarize the demographic characteristics and pain management practices. To assess the “level of awareness” regarding cancer pain management, participants were categorized into groups based on the quartile distribution of their cumulative awareness scores: “Strong awareness” (≥ 75th percentile), “Moderate awareness” (25th–75th percentile), and “Weak/No awareness” (≤ 25th percentile).One-way analysis of variance (ANOVA) was performed to examine differences in pain management perceptions across education levels and other demographic characteristics. Prior to conducting ANOVA, the normality of the data was verified using the Kolmogorov-Smirnov test, and homogeneity of variances was confirmed via Levene’s test to ensure the statistical assumptions were met.To control for potential confounding variables and identify independent predictors of management awareness, a multinomial logistic regression analysis was employed. Odds ratios (ORs) with 95% confidence intervals (CIs) were calculated to estimate the strength of these associations.For the questionnaire’s reliability and validity, Cronbach’s alpha coefficients were calculated. Exploratory factor analysis (EFA) with varimax rotation was used to determine the factor structure, with sampling adequacy assessed via the Kaiser-Meyer-Olkin (KMO) measure and Bartlett’s test of sphericity. All statistical tests were two-tailed, and a p$-value < 0.05$ was considered statistically significant.

### Quality control

Multiple measures were undertaken to ensure research quality. The questionnaire was reviewed by clinical and methodological experts to ensure content validity. Survey administrators were trained to standardize data collection procedures. Double data entry and cross-validation were performed to minimize input errors. Statistical analyses were conducted by experienced biostatisticians to enhance analytical reliability.

## Results

### Participant regional distribution and characteristics of physicians

#### Participant regional distribution

**Fig. 1:**
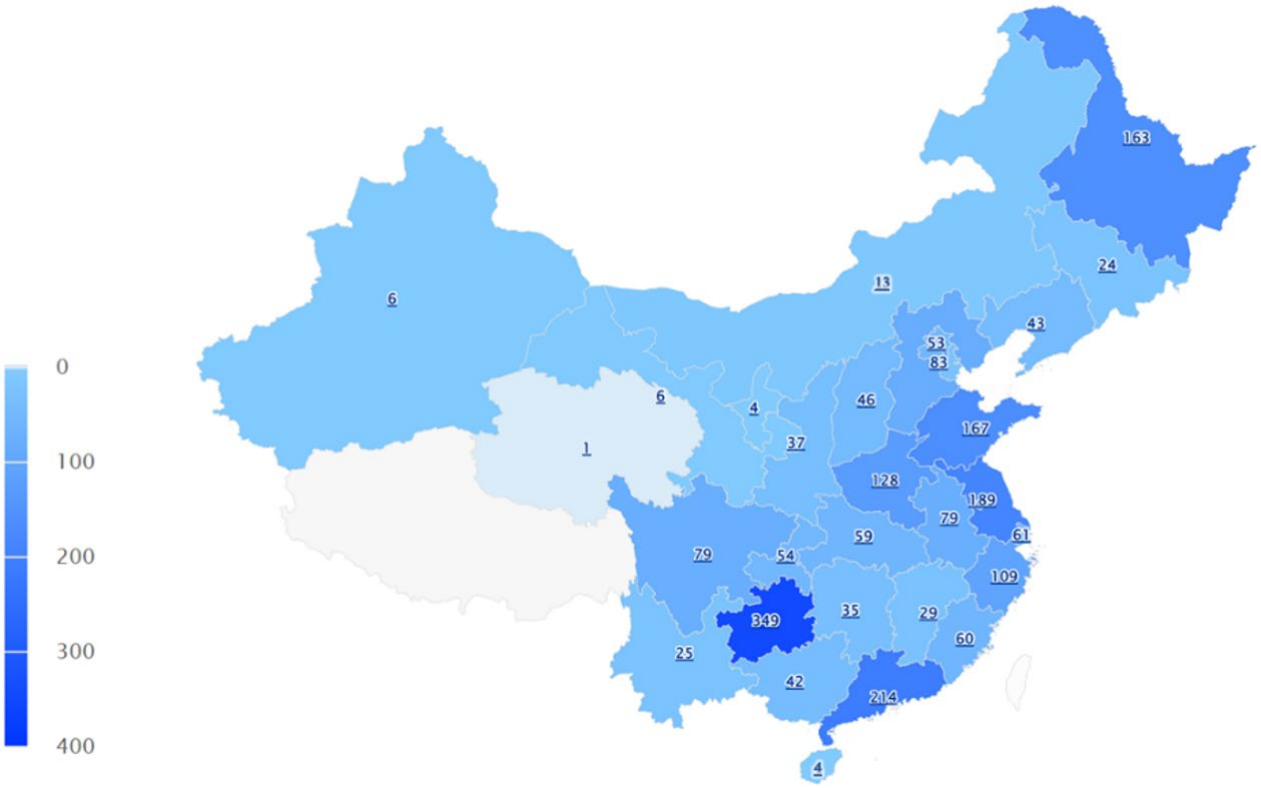
China map highlighting the geographic distribution of physician respondents in China.

This map visualizes the regional distribution of 2,188 physicians who participated in a nationwide survey on cancer pain management practices(Fig. 1). Each province in China is shaded based on the number of respondents, with darker tones indicating higher participation. While the broader global context is shown, data collection was limited to mainland China.

A total of 2,188 valid questionnaires were collected from September 1, 2019, to September 30, 2024. The physician sample was demographically diverse and predominantly young. Slightly more respondents were male (53.66%) than female (46.34%). Most participants had less than five years of work experience, with 28.79% reporting less than one year. The largest age group was 23–30 years (31.76%). The majority were employed in tertiary hospitals (35.42%) and affiliated with oncology (27.15%) or pain management (25.50%) departments. Most were junior-level professionals (50.60%) and held a Bachelor of Medicine (61.88%), followed by Master’s (22.26%) and Doctoral degrees (10.15%). These findings indicate that the study population was composed primarily of well-educated, early-career physicians with direct exposure to cancer-related clinical care (Table 1).

**Table 1:** Characteristics of Physicians(*n* = 2,188).

Characteristic	*n* (%)
Gender	
Female	1014(46.34)
Male	1174(53.66)
Years of work	
< 1 year	630(28.79)
1–3 years	406(18.56)
3–5 years	435(19.88)
5–10 years	443(20.25)
> 10 years	274(12.52)
Age	
<18years	3(0.10)
18–23 years	575(26.41)
23–30 years	695(31.76)
30–45 years	642(29.34)
> 45 years	273(12.48)
Hospital classification	
Tertiary levels of general hospitals	775(35.42)
Primary levels of general hospitals	412(18.83)
Secondary levels of general hospitals	639(29.20)
Cancer specialist hospitals	362(16.55)
Department of hospital	
Internal Medicine department	423(19.33)
Surgery Department	365(16.68)
Pain department	558(25.50)
Oncology department	594(27.15)
Others	248(11.34)
Professional title	
Junior professional title	1107(50.60)
Intermediate professional title	741(33.87)
Senior professional title	93(4.25)
Others	247(11.28)
Educational background	
Bachelor of Medicine, MB	1354(61.88)
Master of Medicine, MM	487(22.26)
Doctor of Medicine, PhD	222(10.15)
Others	125(5.71)

Note: MB = Bachelor of Medicine; MM = Master of Medicine; PhD = Doctor of Philosophy in Medicine.

### Cognition of cancer pain

#### Understanding of pain definitions and awareness of pain management

**Fig. 2:**
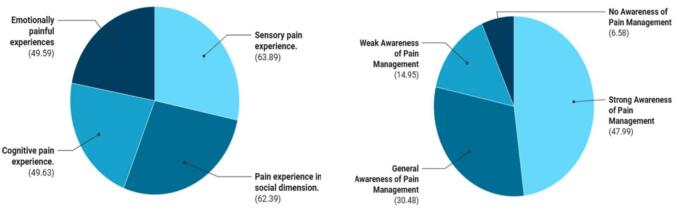
Diverse pain experiences and varying levels of management awareness.

Respondents demonstrated a multidimensional understanding of cancer pain. A majority recognized pain as a sensory (63.89%) and social (62.39%) experience, followed by emotional (49.59%) and cognitive (49.63%) components. Approximately 78% accurately defined cancer pain as originating from the malignancy or its treatment. Despite this, practical implementation of pain assessment was often delayed or incomplete due to time constraints in clinical settings. Notably, 6% of respondents misunderstood cancer pain, confusing it with other types of pain When asked about departmental awareness of standardized pain management protocols, 47.99% rated it as strong, 30.48% as moderate, 14.95% as weak, and 6.58% reported no awareness(Fig. 2).

#### Definition of pain and pain assessment methods

Table 2 summarizes physicians’ perceptions of pain and preferred assessment tools. Most respondents identified pain as both a sensory (63.89%) and social (62.39%) experience, while nearly half recognized emotional (49.59%) and cognitive (49.63%) aspects.

**Table 2:** Definition of Pain and Pain Assessment Methods.

Survey content	Specific Items	Proportion
Definition of Pain	Sensory painful experience	63.89%
	Social - dimensional painful experience	62.39%
	Emotional painful experience	49.59%
	Cognitive painful experience	49.63%
Pain Assessment Methods	Verbal Rating Scale (VRS)	30.48%
	Numeric Rating Scale (NRS)	26.51%
	Visual Analogue Scales (VAS)	22.90%
	Facial Expression Pain Rating Scale	12.84%
	WHO Pain Grading Standard	7.27%

Regarding assessment methods, the Verbal Rating Scale (VRS) was the most frequently used (30.48%), followed by the Numeric Rating Scale (NRS, 26.51%) and Visual Analogue Scale (VAS, 22.90%). Less frequently adopted tools included the Facial Expression Pain Scale (12.84%) and WHO pain classification system (7.27%).

### Pain assessment, relief methods, and medication administration preferences

Pain assessment was primarily based on patients’ self-reports (44.42%) and healthcare staff evaluations (36.24%), with family member input playing a minor role (17.09%). Pharmacological analgesia was the predominant pain relief method (73.90%), followed by non-pharmacological approaches such as transcutaneous electrical nerve stimulation (TENS, 67.32%) and physical therapy (64.85%). Acupuncture (38.21%) was less commonly used. In terms of medication administration routes, oral delivery was most preferred (32.91%), followed by intramuscular (27.97%) and intravenous injections (20.38%). Rectal (11.93%) and subcutaneous (6.81%) routes were least favored(Table 3).

**Table 3:** Pain assessment, relief methods, and medication administration preferences.

Investigation dimension	Options	Proportion
Subject of pain assessment	The patient themselves	44.42%
	Medical staff	36.24%
	Patient’s family members	17.09%
	Others	2.24%
Pain-relief Methods	Drug analgesia	73.90%
	Transcutaneous electrical nerve stimulation (TENS)	67.32%
	Physical analgesia	64.85%
	Acupuncture analgesia	38.21%
	Others	43.42%
Medication administration routes	Oral administration	32.91%
	Intramuscular injection	27.97%
	Intravenous administration	20.38%
	Rectal administration	11.93%
	Subcutaneous injection	6.81%

### ANOVA: Educational Background and Role Cognition

**Fig. 3:**
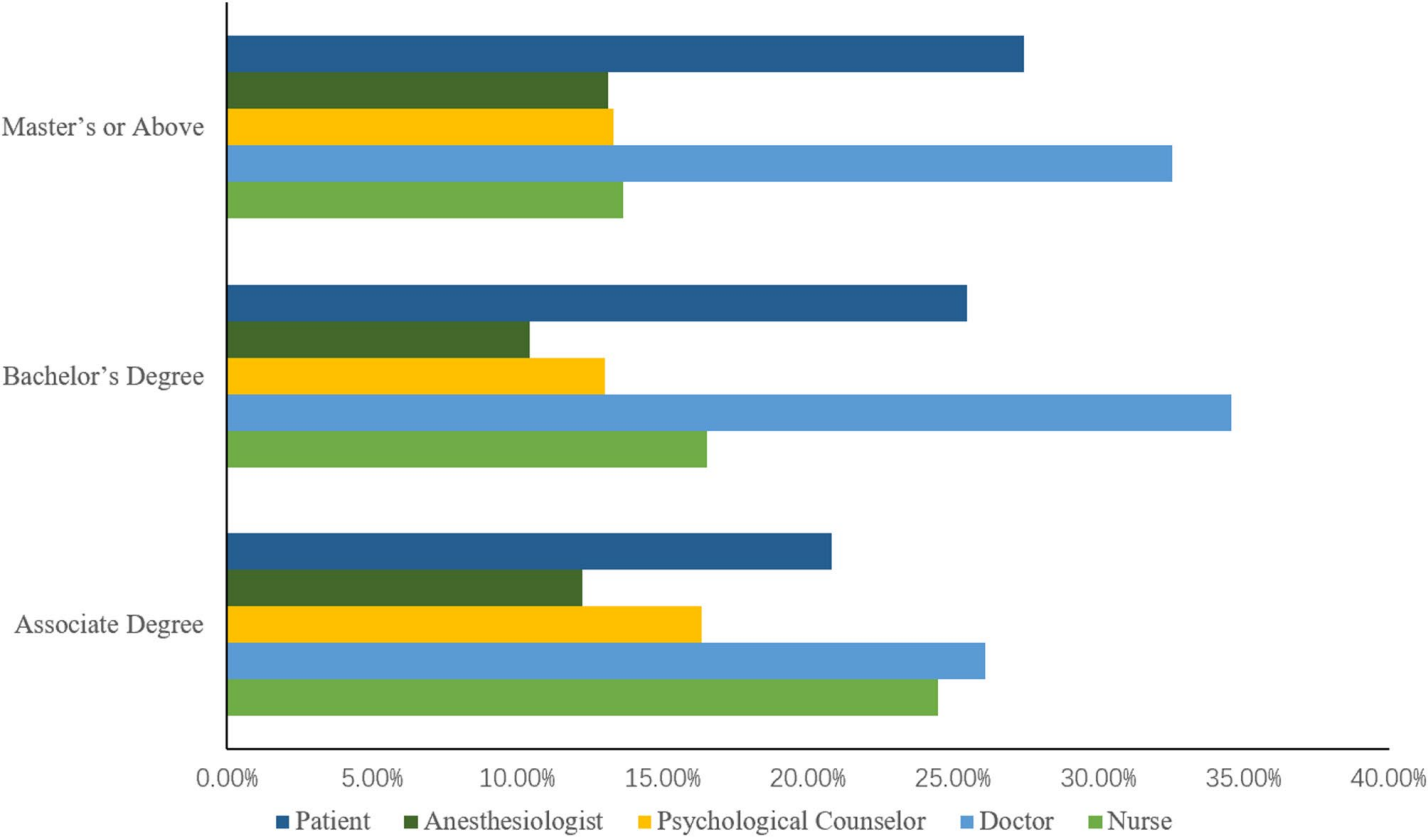
Perceived primary responsible personnel for pain management by education level (%).

One-way ANOVA revealed a significant effect of educational level on respondents’ perception of the main professional responsible for pain management (F = 18.718, *p* < 0.001). Physicians with postgraduate education scored higher (M = 2.74, SD = 0.68) than those with undergraduate (M = 2.23, SD = 0.69) and associate degrees (M = 2.12, SD = 0.67), suggesting a positive association between higher education and role awareness(Table 4; Fig. 3).

**Table 4:** ANOVA of education level and role cognition.

Education Level	*n*	Mean	SD	*P*	F
Associate Degree	247	2.19	0.58		
Bachelor’s Degree	1354	2.43	0.70		
Master’s or above	587	2.72	0.63		
				0.000	62.01

### Validity analysis

Exploratory factor analysis (EFA) extracted 7 factors, explaining 63.26% of the total variance. The Kaiser-Meyer-Olkin (KMO) value was 0.764, and Bartlett’s Test of Sphericity was significant (χ^2^ = 1150.638, df = 136, *P* < 0.001), indicating good sampling adequacy and factorability. Key variables such as “Professional title”, “Age”, and “Clinical working years” demonstrated high communality, supporting construct validity(Table 5).

**Table 5:** Key outputs from exploratory factor analysis.

Metric	Value
Number of extracted factors	7
Cumulative explained variance	63.26%
KMO measure	0.764
Bartlett’s Test (χ^2^)	1150.638
Degrees of freedom (df)	136
p-value	< 0.001

### Correlation analysis

Pearson correlation analysis(Table 6) showed a moderate positive correlation between “Rotation in oncology department” and “Awareness of standardized pain management in your department” (*r* = 0.67), suggesting that clinical exposure in oncology may enhance awareness of pain management protocols. In contrast, “Educational background” and “Source of pain assessment criteria” demonstrated a weak and non-significant negative correlation (*r* = -0.03).

**Table 6:** Correlation matrix.

Variable A	Variable B	*r*	Significance
Rotation in oncology department	Awareness of departmental pain management	0.67	*p* < 0.01
Educational background	Source of pain assessment criteria	-0.03	NS

### Multinomial logistic regression analysis and correlation analysis of management intensity

The results of the multinomial logistic regression analysis exploring factors associated with different levels of pain management awareness (“Strong Management Awareness,” “Moderate Management Awareness,” and “Weak Management Awareness”) showed that, in terms of age, using the group of > 45 years as the reference category, the impact of age groups 18–23 years, 23–30 years, and 30–45 years on different levels of pain management awareness was mostly not significant. For example, for the 18–23 years group, the regression coefficient was B = 0.217, odds ratio (OR) = 1.242 (95% CI: 0.699–2.205), and *P* = 0.460. In terms of educational background, compared with the reference group (presumably those with a junior college education or below), individuals with a junior college degree were less likely to have strong pain management awareness (B = -0.416, OR = 0.660, 95% CI: 0.373–1.168, *P* = 0.154), while those with a bachelor’s degree or higher showed a slight increase in the likelihood, though not significant (B = 0.191, OR = 1.210, 95% CI: 0.806–1.816, *P* = 0.357). Regarding professional titles, compared to those with titles of Associate Chief Physician and above, interns, standardized training physicians, and resident physicians were less likely to exhibit strong management awareness, with some results being statistically significant. For instance, interns had a lower likelihood of strong management awareness (B = -2.050, OR = 0.129, 95% CI: 0.017–0.951, *P* = 0.044). As for years of work experience, using > 10 years as the reference group, those with 5–10 years of work experience showed a positive and significant correlation with strong pain management awareness (B = 0.803, OR = 2.233, 95% CI: 1.199–4.161, *P* = 0.011). In terms of medication choices, the use of non-opioid drugs was negatively correlated with strong pain management awareness (B = -0.919, OR = 0.399, 95% CI: 0.267–0.597, *P* = 0.000), while the use of weak opioids was positively correlated with strong awareness (B = 2.067, OR = 7.903, 95% CI: 2.407–25.947, *P* = 0.001). Similar trends were observed for moderate and weak pain management awareness. This study used “No Pain Management Awareness” as the reference category, and redundant parameters were set to zero (Table 7).

**Table 7:** Multinomial logistic regression analysis and correlation analysis of management intensity.

Pain Management Awareness*	Strong management awareness			Moderate management awareness			Weak management awareness		
	B	OR(95%CI)	*P*	B	OR(95%CI)	*P*	B	OR(95%CI)	*P*
Age									
18–23 years	0.217	1.242(0.699 ~ 2.205)	0.460	-0.255	0.775(0.43 ~ 1.395)	0.396	-0.242	0.785(0.414 ~ 1.488)	0.458
23–30 years	0.396	1.485(0.836 ~ 2.638)	0.177	0.069	1.071(0.597 ~ 1.922)	0.817	-0.038	1.031(0.570 ~ 1.864)	0.906
30–45 years	0.374	1.454(0.813 ~ 2.601)	0.207	0.031	1.031(0.570 ~ 1.864)	0.920	-0.076	1.031(0.570 ~ 1.864)	0.816
>45years	0#			0#			0#		
Educational background									
Junior college	-0.416	0.660(0.373 ~ 1.168)	0.154	-0.391	0.677(0.375 ~ 1.220)	0.194	-0.081	0.979(0.526 ~ 1.821)	0.946
Bachelor’s degree and above	0.191	1.210(0.806 ~ 1.816)	0.357	-0.030	1.031(0.678 ~ 1.566)	0.887	-0.22	0.871(0.553 ~ 1.372)	0.552
Master’s degree and above	0#			0#			0#		
Professional Title									
Intern Physician	-2.050	0.129(0.017 ~ 0.951)	0.044	-2.131	0.119(0.016 ~ 0.889)	0.038*	-1.792	0.167(0.021 ~ 1.314)	0.089
Standardized Training Physician	-2.224	0.108(0.015 ~ 0.800)	0.029*	-2.128	0.119(0.016 ~ 0.892)	0.038*	-1.828	0.161(0.020 ~ 1.268)	0.083
Resident Physician	-1.558	0.211(0.027 ~ 1.621)	0.135	-1.498	0.224(0.029 ~ 1.748)	0.153	-1.293	0.275(0.033 ~ 2.266)	0.230
Attending Physician	-1.460	0.232(0.030 ~ 1.821)	0.165	-1.179	0.308(0.039 ~ 2.446)	0.308	-1.439	0.237(0.028 ~ 2.007)	0.187
Associate Chief Physician and above	0#			0#			0#		
Years of work									
<1 year	0.334	1.397(0.831 ~ 2.347)	0.207	-0.191	0.826(0.486 ~ 1.406)	0.481	-2.227	0.979(0.447 ~ 1.420)	0.441
1–3 years	0.257	1.293(0.727 ~ 2.298)	0.382	-0.031	0.970(0.540 ~ 1.742)	0.919	-0.11	0.896(0.474 ~ 1.693)	0.734
3–5 years	0.017	1.017(0.572 ~ 1.807)	0.955	-0.373	0.689(0.382 ~ 1.243)	0.216	-0.198	0.820(0.434 ~ 1.549)	0.542
5–10 years	0.803	2.233(1.199 ~ 4.161)	0.011**	-0.366	1.442(0.765 ~ 2.719)	0.257	-0.207	1.230(0.621 ~ 2.438)	0.553
>10 years	0#			0#			0#		
Medication Choices									
Non-Opioids	-0.919	0.399(0.267 ~ 0.597)	0.000***	-0.709	0.492(0.324 ~ 0.747)	0.001***	-0.445	0.641(0.408 ~ 1.005)	0.053
Weak Opioids	2.067	7.903(2.407 ~ 25.947)	0.001**	2.246	9.449(2.859 ~ 31.233)	0.000***	1.651	5.212(1.522 ~ 17.849)	0.009**
Strong Opioids	0#			0#			0#		

*The reference category is: no awareness of pain management, #In regression analysis, when a parameter is redundant, it is set to zero. “Note: *p < 0.05, **p < 0.01, ***p < 0.001.

### Results of in-depth interviews

Ten frontline physicians participated in semi-structured interviews. Of them, 60% rated their understanding of cancer pain as “good” or “average,” while 30% admitted to having poor knowledge. Most respondents (70%) relied primarily on pharmacological treatment, with only a few incorporating psychological support or multimodal approaches.

All participants expressed the need for further training, particularly in pharmacology, integrative strategies, and psychological care. Notably, 90% acknowledged the importance of multidisciplinary collaboration in improving cancer pain outcomes. These findings complement the quantitative results and emphasize unmet training needs and systemic gaps in practice(Table 8).

**Table 8:** The in-depth interview results of 10 first-line clinical doctors in China on cancer pain.

Doctor ID	Age	Gender	Title	Awareness of cancer pain	Main management strategy	Training needs	View on multidisciplinary collaboration
1	35	Male	Attending Physician	Good	Drug therapy as primary	Need more training	Very important
2	42	Female	Associate Chief Physician	Fair	Drug-based	Has demand	Important
3	29	Male	Physician	Poor	Main drugs	Strong demand	Important
4	50	Female	Chief Physician	Good	Drug + psychological counseling	Need training	Important
5	38	Male	Physician	Fair	Drug therapy as primary	Need training	Important
6	33	Female	Attending Physician	Good	Comprehensive therapy	Has demand	Very important
7	45	Male	Associate Chief Physician	Good	Drug-based	Need training	Important
8	31	Female	Physician	Fair	Drug-based	Strong demand	Important
9	40	Male	Attending Physician	Poor	Drug + physical therapy	Has demand	Very important
10	37	Female	Physician	Good	Comprehensive treatment	Need more training	Important

## Discussion

This study surveyed physicians’ knowledge and management of cancer pain across 29 provinces in mainland China, revealing several notable findings.

First, the regional distribution of respondents revealed significant disparities. While substantial responses were collected from both economically underdeveloped areas such as Guizhou and more developed provinces like Guangdong and Shandong, participation from several remote western regions^20–22^. Strengthening training and policy support in these areas is essential to promote equitable pain care. The regional disparities observed in this study, with lower awareness and participation in certain western provinces, are deeply rooted in macro-level systemic factors^23^. Specifically, the uneven distribution of health resources in China often concentrates specialized pain centers in eastern urban hubs. Moreover, educational opportunities for standardized opioid titration are less accessible in remote areas. These gaps are further compounded by policy-level inequalities, such as varying local implementation of ‘Pain-Free Ward’ initiatives, which underscores the need for centralized policy support to ensure equitable cancer pain care across mainland China^24–26^.

Most participants were young physicians with relatively high educational backgrounds but limited clinical experience, mainly working in oncology and pain departments at tertiary hospitals. Although their motivation and clinical exposure provide a good foundation, limited experience may hinder management of complex cases^27,28^. Emphasizing practical training and multidisciplinary collaboration is necessary, particularly in primary and secondary hospitals^29,30^.

Physicians generally demonstrated a multidimensional understanding of cancer pain, recognizing sensory, social, emotional, and cognitive components, and correctly identified cancer pain’s origin. Nonetheless, a minority exhibited misunderstandings, and pain assessment was often delayed due to clinical workload, indicating a gap between knowledge and practice^31,32^. Clinical workflows should be optimized to integrate pain assessment routinely^33^. Beyond the identified gap, our qualitative data suggests a deeper communicative barrier: the omission of the explicit word ‘pain’ during consultations^34,35^. This phenomenon may be attributed to an emotional defense mechanism, where physicians subconsciously distance themselves from the patient’s suffering to mitigate personal burnout. Additionally, the institutional hospital culture in China, which traditionally prioritizes objective clinical indicators over subjective patient experiences, may inadvertently discourage a direct dialogue about pain^36^.

Regarding assessment tools, simple subjective scales such as the Verbal Rating Scale, Numeric Rating Scale, and Visual Analogue Scale were preferred, fitting busy clinical environments^37–39^. More comprehensive tools like the Facial Expression Pain Scale and WHO classification were less used, highlighting the need for wider adoption through training^12,40^.

Pharmacological treatment remained the primary pain relief method, with oral administration favored. Non-pharmacological therapies, including transcutaneous electrical nerve stimulation and physical therapy, were increasingly accepted, while traditional methods like acupuncture were less common, suggesting further integration efforts are needed^41–43^.

Multivariate analysis revealed that educational level, professional title, and clinical experience were significantly associated with physicians’ awareness of pain management responsibilities; higher education and greater experience may contribute to stronger awareness^44–46^. This underscores the importance of continuing education and professional development. Additionally, medication choices correlated with management awareness, emphasizing the need for rational analgesic use in training programs. To further contextualize these results, it is essential to consider the broader East Asian cultural context. Our findings regarding medication caution mirror challenges observed in Japan and South Korea, where ‘opiophobia’ and the cultural value of ‘stoicism’ remain significant barriers^34^. Studies in these neighboring countries have similarly identified that patients often under-report pain to avoid being ‘burdensome,’ and clinicians remain cautious about the stigma of opioid addiction^47^. This cross-cultural consistency reinforces the need for pain management strategies tailored to East Asian socio-cultural values that prioritize endurance. Several limitations must be acknowledged. First, the cross-sectional design prevents the establishment of causal relationships between physician characteristics and pain awareness. Furthermore, as a cross-sectional study, these findings cannot establish causal relationships between physician characteristics and pain awareness. The results should be interpreted as exploratory rather than definitive evidence for policy changes. Second, as data were collected via self-report, results may be subject to social desirability bias, where physicians might overstate their adherence to guidelines. Third, our sample predominantly consisted of younger physicians in tertiary hospitals. While these individuals represent a key force in oncology, our findings may not be fully generalizable to senior clinicians or those practicing in primary care settings^48^.

In-depth interviews confirmed that pharmacological treatment dominated clinical practice, with limited psychological support and multidisciplinary collaboration. Participants expressed a strong demand for systematic training, especially in pharmacology, integrative approaches, and psychological care, underscoring the need to strengthen multidisciplinary teams and comprehensive management strategies^42,49^. This need is further elucidated through data triangulation within our explanatory sequential design^5^. While the quantitative survey indicated high theoretical awareness of multidimensional pain, the qualitative interviews revealed that systemic constraints—such as time pressure and a lack of structured multidisciplinary protocols—prevent this awareness from becoming action^50,51^. This integration allows us to confirm that the clinical gaps are not merely due to individual ignorance but are rooted in structural barriers within the healthcare system. While multidisciplinary care is widely recognized as essential, its implementation requires a standardized framework. We recommend the establishment of ‘Integrated Cancer Pain Clinics’ where oncologists, pain specialists, and clinical pharmacists conduct joint assessments. Furthermore, psychological support should be formally integrated into pain rounds, and digital multidisciplinary team (MDT) platforms should be leveraged to facilitate communication between specialties in resource-limited settings^50,52^.

In conclusion, this study highlights current gaps in cancer pain knowledge and management among Chinese physicians. Addressing resource disparities, enhancing targeted education, and fostering multidisciplinary collaboration could potentially improve standardized pain management and may lead to better patient quality of life.

## Supplementary Information

Below is the link to the electronic supplementary material.

Supplementary Material 1

## Data Availability

The datasets generated and/or analyzed during the current study are available from the corresponding author on reasonable request.
